# Supplementary material for: Patterns of Intron Gain and Loss in Fungi
Source: PLoS Biol. 2004 Nov 30;2(12):e422. doi: 10.1371/journal.pbio.0020422 (PMC532390; doi:10.1371/journal.pbio.0020422)
Supplement: Table S1 — Also available at http://genes.mit.edu/NielsenEtAl/. (4.3 MB ZIP). [file pbio.0020422.st001.zip › NielsenEtAl/html/475.html]

AN8831.1.NCU01069.1.MG05528.1.FG02092.1


```
 CLUSTAL W (1.82) Multiple Sequence Alignments - Introns Inserted


Sequence 1: NCU01069.1	323 aa
Sequence 2: MG05528.1	273 aa
Sequence 3: FG02092.1	261 aa
Sequence 4: AN8831.1	272 aa
Alignment Length: 323 aa
Number Identitical Residues: 216 aa
Alignment Score (without introns) 8526


MG05528.1 	---------------------------------MDMHPG~IARTNTP---------~---
NCU01069.1	MEAVELRFCTCHGLLAARWDFVAKAFVAWQVDPADVVQD~YAQSHSKERVILAGSA1NGF
FG02092.1 	---------------------------------------~----------------~---
AN8831.1  	---------------------------------MSWAGA2FTLTCVLS--------~---
          	                                  .      : :   .            

MG05528.1 	-----CLAG~FKKNVNRATTQVMMKTG1HVEKTNDRDYEVEER~RFRTMEAASNRLQKEA
NCU01069.1	RLFRLCLTG~FKKNVNRATTQVMMKTG~HVEKTNDRDYEVEER2RFKTMEAASLRLQKEA
FG02092.1 	----MSWAG1FKKNVGRATTQVMMKTG1HVEKTNDRDYEVEER2RFKTMEAAALRLQKES
AN8831.1  	-------AG~FKKNVNRATTQVMMKTG1HVERTNDRDYEIEER2RYRTMEAAANRLQKEA
          	       :* *****.*********** ***:*******:*** *::*****: *****:

MG05528.1 	KGYLDSLRA~MTASQMRIAETIDAFYGDSGAKDGVSRSYKQAVEDLDAETIKALDGPYR~
NCU01069.1	KGYLDSLRA1MTASQMRIAETIDAFYGEAGAKDGVSKSYKQAVEDLDAETIKALDGPYR~
FG02092.1 	KGYLDSLRA1MTASQMRIAETIDAFYGDSGAKDGVSRSYKQAVEDLDAETIKALDGPYR~
AN8831.1  	KGYLDSLRA1MTASQMRIAETIDAFYGDAGAKDGVSRSYKQAVEDLDAETIKALDGPYR2
          	********* *****************::*******:********************** 

MG05528.1 	TTVLEPITRFCNYFPDVNEC~IKKRGHKLLDYDALRARVKKLAEKPDKDVTKLPRTEKEM
NCU01069.1	TTVLDPITRFCAYFPDVNEC1IKKRGHKLLDYDALRAKVKKLVEKPDKDVTKLPRAEKEM
FG02092.1 	TTVLDPITRFCAYFPDVNEA~IKKRAHKLLDYDALRAKVKKLAEKPDKDATKLPRTEKEM
AN8831.1  	TTVLDPISRFCAYFPDVNEC~IKKRNNKLLDYDAMRAKVKRLVEKPDKDATKLPRTEREL
          	****:**:*** *******. **** :*******:**:**:*.******.*****:*:*:

MG05528.1 	DMSRAAYEQLNEQLCTELPQLIDLRVPYLDPSFEALVKIQLRFCAEAYSRMAQVQQYLDA
NCU01069.1	EMAKAAYEQLNEQLCTELPQLIDLRVPYLDPSFEALVKIQLRFCAEAYSRMAQVQQYLDP
FG02092.1 	EMAKAAYEQLNEQLSTELPQLIELRVPYLDPTFEALVKIQLRFCAEAYSRMAQVQQYLDA
AN8831.1  	EMAKQAYEQLNEQLFTELPQLIDLRVPYLDPSFEALVKIQLRFCAEAYSRMAQVQQYLDA
          	:*:: ********* *******:********:***************************.

MG05528.1 	DTRDQYAQGHLDSRVEQVLQEIRELSISGTV
NCU01069.1	DTREQYAQGQLDTRVEQVLQEIRELSISGTV
FG02092.1 	DTRDQYAEGQLDARVEQVLQEIRELSISGTV
AN8831.1  	ETRDQYARGDLDNRVEEVLQEIRDLSIAGTV
          	:**:***.*.** ***:******:***:***
```
